# Cost-effectiveness of comprehensive preventive measures for coal workers’ pneumoconiosis in China

**DOI:** 10.1186/s12913-022-07654-7

**Published:** 2022-02-28

**Authors:** Xiaoyan Ge, Kai Cui, Honglin Ma, Siqi Zhao, Weihan Meng, Wenbo Wang

**Affiliations:** Department of Health Statistics, School of Public Health, Jinzhou Medical University, No. 40 Songpo Road, Jinzhou, Liaoning, 121000 People’s Republic of China

**Keywords:** Coal workers’ pneumoconiosis, Health economics, Cost-effectiveness, Comprehensive measures

## Abstract

**Background:**

Coal workers’ pneumoconiosis (CWP) remains one of the most severe occupational diseases in China. Despite the implementation of CWP comprehensive preventive measures, the unreasonable allocation of investment by coal enterprises limits the effect of preventing CWP, especially when the health resources are inadequate. This study aims to evaluate the cost-effectiveness of comprehensive measures for CWP from the perspective of coal enterprises.

**Methods:**

Comprehensive measures and two primary interventions (engineering controls and individual protective equipment) were selected. A time-dependent Markov model was developed to evaluate cost and quality-adjusted life-years (QALYs). The input data were collected from the survey and literature. A hypothetical null situation, in which the currently implemented interventions were eliminated, was used as a comparator based on the generalised cost-effectiveness analysis (GCEA) recommended by the World Health Organization (WHO). The primary outcomes of the model were reported in terms of incremental cost-effectiveness ratios (ICERs). Uncertainty was verified using one-way and probabilistic sensitivity analyses.

**Results:**

The QALYs of the comprehensive measures, engineering controls, and individual protective equipment were 17.60, 17.50, and 16.85 years, respectively. Compared with null, the ICERs of the interventions were 65,044.73, 30,865.15, and 86,952.41 RMB/QALY, respectively. Individual protective equipment was dominated by an ICER of -11,416.02 RMB/QALY compared to engineering controls. Sensitivity analysis suggested the robustness of the results.

**Conclusions:**

The comprehensive preventive measures for CWP that are currently implemented in Chinese state-owned mines are cost-effective. In comprehensive measures, engineering controls are more cost-effective than individual protective equipment. Investment in engineering controls should be increased to improve the cost-effectiveness of preventing CWP.

**Supplementary Information:**

The online version contains supplementary material available at 10.1186/s12913-022-07654-7.

## Background

Coal workers’ pneumoconiosis (CWP) is a common occupational disease caused by the long-term inhalation of coal mine dust during mining [1, 2]. CWP is an incurable disease because of its irreversible progression. In the USA, federal standards were enacted in 1969 to establish primary and secondary prevention programs for coal miners [3]. However, studies have shown a marked resurgence and increasing severity of CWP over the past 20 years [3–5]. Since the 1950s, comprehensive measures, including engineering and technical interventions for decreasing coal dust, individual protection interventions, and management, have been adopted in state-owned coal mines in China. However, CWP remains one of the most severe occupational diseases in China. From 2011 to 2018, 12,000–16,000 new cases of CWP have been reported in China each year. In 2019, the State Council of China enacted the Health China Initiative (2019–2030) and the Action Plan for Prevention and Treatment of Pneumoconiosis to reduce the number of new cases [6–8]. Therefore, prevention and control of CWP will be a crucial issue for occupational health administration departments, and coal enterprises in China in the future.

Comprehensive measures are an integrated and complex strategy for preventing CWP, and a large amount of investment from coal enterprises is required. Local governments and coal enterprises pay more attention to financial benefits and avoid production accidents. This leads to an unreasonable allocation of investment. To balance the CWP prevention costs with financial benefits, coal enterprises need to devise strategies to rationally allocate limited health resources. Recently, several health economics studies in developed countries have mainly focused on skeletal muscle diseases and occupational stress [9–12]. Few studies have focused on the cost-effectiveness of preventive measures for CWP in coal enterprises. Most economic studies on CWP in China have evaluated direct or indirect economic losses from the patient’s perspective [13, 14]. To the best of our knowledge, few studies have analysed the cost-effectiveness of interventions for CWP from the perspective of coal enterprises.

In this study, we evaluated the cost-effectiveness of comprehensive measures against CWP from an enterprise perspective. We also compared the cost-effectiveness of the primary interventions in comprehensive measures. Our research will provide information for coal enterprises and occupational health administration departments to optimise the allocation of limited health resources.

## Methods

### Study design

This study compared the cost-effectiveness of three interventions for preventing CWP using the Markov model, with a hypothetical null scenario as a comparator. Cost data were collected from six coal mines in China. The transition probabilities of the model were obtained from our previous study, as well as the literature. A cost-effectiveness analysis was conducted from the perspective of coal enterprises.

### Modelling

**Fig. 1:**
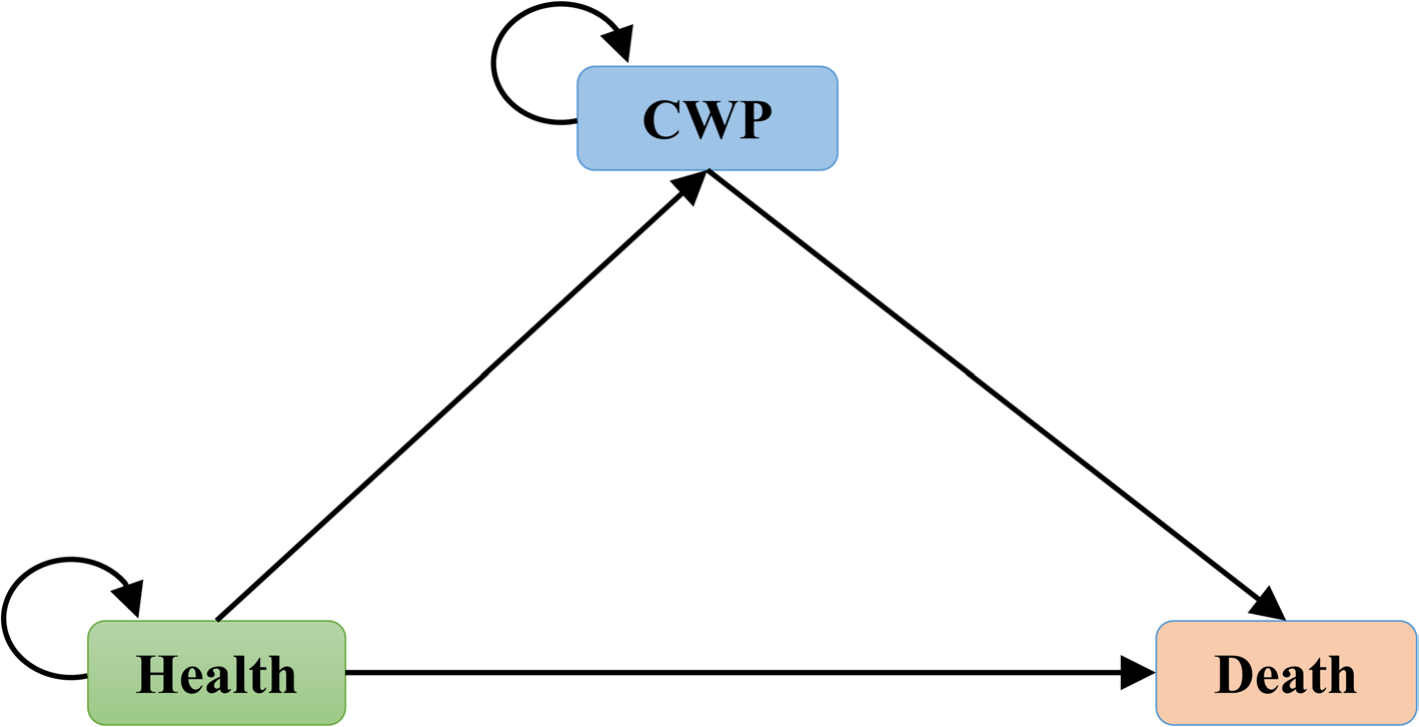
Diagram of the three-state Markov model. Arrows represent possible transitions through the health states

A three-state time-dependent Markov model was constructed to simulate the process of CWP in a hypothetical cohort of coal miners aged ≥ 20 years. The model comprised three states: health, CWP, and death. We assumed that healthy miners aged 20 years entered the model and switched between states with the cycle running. Death was used as the terminal state. The number of cycles ended after 43 years. Details of the model are presented in Fig. 1. The Markov model was constructed using TreeAge Pro 2011 (TreeAge Software, Williamstown, MA, USA).

### Interventions

Comprehensive measures, engineering controls, and individual protective equipment were selected for this study. The latter two were the primary interventions in comprehensive measures, that could directly affect the inhalation of coal dust. Engineering controls referred to dustproof engineering technology and devices, including water stemming blasting, wetting a drilled or ground surface, spraying, air curtains, water curtains, ventilation systems, among others. Individual protective equipment included dust masks, respirators, caps, helmets, garments, gloves, among others. Comprehensive measures referred to the combination of the two primary interventions, occupational health administration, supervision, health surveillance, and other measures. Comprehensive measures represent the currently implemented interventions in Chinese state-owned coal mines.

### Comparator

Based on the generalised cost-effectiveness analysis (GCEA) recommended by the World Health Organization (WHO), the cost-effectiveness of interventions was compared with a “null” scenario [15]. The null scenario was a hypothetical situation in which the currently implemented interventions were eliminated. In this study, it was quantified by removing the costs and effects of the two primary interventions in the current comprehensive measures.

### Costs

In this study, the costs were considered as the investment for preventing CWP for each miner by the coal enterprise. The costs were reported in the Chinese Renminbi (RMB, official currency of China) in 2020. We surveyed the costs of six coal mines in Shanxi Province, China. All the mines were state-owned, with annual coal production of over 1.2 million tons. The costs included the annual expenses of acquisition, operation, maintenance, repair of engineering devices, annual expenses of acquisition, maintenance, replacement of the individual protective equipment, and occupational health management costs. The costs of management included the annual expenditures of occupational health training for coal miners, setting the warning signs, dust detection, annual income of the occupational health management staff, and expenses for health surveillance for the miners. According to the survey, the total yearly costs among the six coal mines ranged from 5.43 to 14.75 million RMB. The per capita yearly cost was calculated using the total cost divided by the number of underground coal miners, and it ranged between 3,285.89 and 11,032.07 RMB. The details of these costs are listed in Table 1.

**Table 1:** Annual costs of CWP preventive measures in six coal mines

**Costs**		**Range**	$\overline{{\varvec{x}}}\pm {\varvec{s} }$
Total costs (million RMB)		5.43–14.75	10.80 ± 3.90
Per capita costs (RMB)	Engineering controls	429.63–3352.50	1582.80 ± 1317.62
	Individual protective equipment	441.14–6572.36	2486.25 ± 2537.75
	Occupational health management	990.00–4768.80	2477.76 ± 1456.04
	Total	3285.89–11,032.07	6546.81 ± 2522.37

### Transition probabilities

#### From health to CWP

The time-dependent transition probabilities from health to the CWP state in comprehensive measures were collected from our previous study of a historical cohort of coal miners who were exposed to coal dust in four state-owned coal enterprises in China between 1970 and 2013 [16, 17]. During this period, comprehensive measures were adopted in coal mines in China. These four enterprises have high annual coal yields in China’s coal industry, and similar processes in mining techniques. These four enterprises are representative of the coal mining industry in China, as far as the experience in mining processes and dust-proofing efforts are concerned. Briefly, the working history of 87,904 miners was collected, and 2,873 miners developed CWP [16, 17]. The life-table method was adopted to analyse the data, and the transition probability was estimated using the formula given below [18]:

$$\widehat{P}\left({t}_{0},t\right)=I/[{N}_{0}^{^{\prime}}-\left(W\left/ 2\right.\right)]$$

where $\widehat{P}\left({t}_{0},t\right)$ is the transition probability through the period (*t*_*0*_*, t*); *I* denotes the number of new cases; *W* is the number of withdrawals; and ${N}_{0}^{^{\prime}}$ is the number of disease-free individuals at time *t*_*0*_.

If the current intervention mix comprises purely preventive interventions, only the incidence of disease is affected when their effect is removed [15]. Therefore, we assumed that eliminating engineering controls and individual protective equipment only caused a change in the transition probability from health to CWP. The back-adjusting method in GCEA was adopted to assess the transition probabilities in the null scenario and other interventions [19, 20]. The transition probability in the null scenario was calculated using the following formula:

$${\lambda }_{N}=\frac{{\lambda }_{C}}{(1-{c}_{1}\times {e}_{1})(1-{c}_{2}\times {e}_{2})}$$

where *λ*_*N*_ is the transition probability in the null scenario; *λ*_*C*_ is the transition probability of current comprehensive measures; *c*_*1*_ and *c*_*2*_ are the coverages of the two interventions of engineering controls and individual protective equipment, respectively; and *e*_*1*_ and *e*_*2*_ are efficiencies. In this study, coverage for engineering controls was defined as the extent to which the dustproof equipment and devices covered the underground roadway. Efficiency was the proportion to which the intervention decreased coal dust at the workplace. For individual protective equipment, the coverage was represented by the proportion of miners using individual protective equipment, and the efficiency was the extent to which intervention reduced miners’ exposure to respirable coal dust, other than using engineering dustproof controlling measures. After considering the relevant literature and expert opinions, we assumed that *c*_*1*_ and *e*_*1*_ were both 95%. For the individual protective equipment, *c*_*2*_ and *e*_*2*_ were assumed to be 70%.

For either engineering controls or individual protective equipment, the transition probabilities were calculated using the back-adjusting method, as follows:

$${\lambda }_{N}=\frac{{\lambda }_{C}}{(1-c\times e)}$$

where *c* and *e* are the coverage and effectiveness of the intervention, respectively. The transition probabilities from health to CWP for the interventions are listed in Additional File 1.

#### Transition probabilities between other states

The age-specific mortality from the 6^th^ Population Census of China was used as the transition probability from health to death [21]. Since a healthy individual could only transfer to three states, the probability of staying healthy equalled one minus the transition probability from health to CWP minus that from health to death. The probability of transition from CWP to death was extracted from Han’s study, which reported mortality in 495 patients with CWP in a Chinese state-owned coal mine [22]. Patients were aged between 25 and 70 years, with 5-year intervals. To transform the 5-year interval into one year, the following equation was used [18]:

$${\widehat{P}}_{j}=1-{[1-{\widehat{P}}_{j}({t}_{0}, {t}_{j})]}^{1/j}$$

where ${\widehat{P}}_{j}$ is the 1-year transition probability for the *j*^*th*^ time interval; *j* represents the number of equal time intervals; and ${\widehat{P}}_{j}({t}_{0}, {t}_{j})$ is the probability over five years. An equal probability of the 25-age group was assumed for the 20–24 age group. The probability for each year was assumed to be constant within each 5-year interval. The transition probability of staying in the CWP was equal to one minus that from the CWP to death. Details of the transition probabilities are presented in Additional File 2.

### Utilities

Health state utilities are numerical values representing an individual’s preferences for different health-related states, where 0 corresponds to death and 1 corresponds to perfect health [23, 24]. The utilities were multiplied by the time spent in a specific state to produce quality-adjusted life years (QALYs). After an extensive literature review, the utility of Chinese CWP cases was assumed to be 0.666 based on a Chinese study in which 122 pneumoconiosis cases were collected using a stratified sampling method and a survey of quality of life was conducted using the EQ-5D questionnaire.

### Cost-effectiveness analyses

The primary outcomes were cost and QALYs [25]. The incremental cost-effectiveness ratio (ICER), defined as the incremental cost per QALY gained between interventions, was also calculated to identify cost-effective interventions. The intervention was highly cost-effective when the ICER was less than China’s per capita gross domestic product (GDP) in 2020, 72,447 RMB [26]. The intervention was considered cost-effective if it had an ICER between one and three times the per capita GDP and not cost-effective if the ICER exceeded three times the per capita GDP [27]. Cost and QALYs were discounted at an annual rate of 5%, and a half-cycle correction was also applied to the QALYs.

### Sensitivity analyses

One-way and probabilistic sensitivity analyses were performed to explore the impact of variations in key parameters on the outcomes. The costs and utilities were varied by 40% to conduct one-way sensitivity analyses. In probabilistic sensitivity analysis, 10,000 Monte Carlo iterative simulations were performed by random sampling from the distribution of the model input in each iteration. The gamma distribution was assigned to costs, with a beta distribution to utilities. Cost-effectiveness acceptability curves were generated to present the probability of the intervention being cost-effective across a range of willingness-to-pay (WTP) thresholds.

## Results

### Base case analyses

**Fig. 2:**
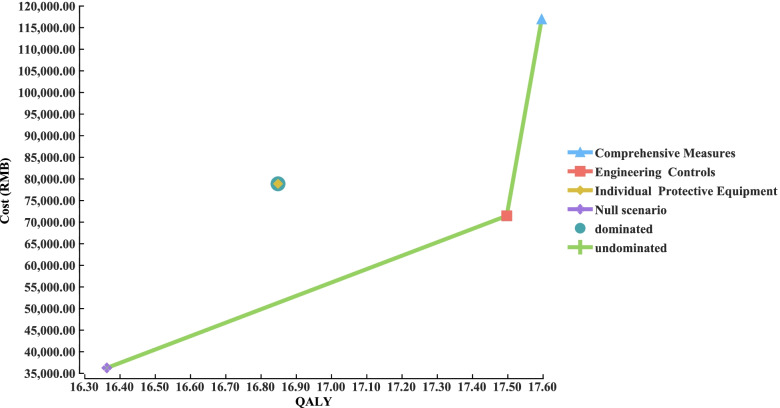
Cost-effectiveness frontier for different interventions

The cost of engineering controls was the lowest, followed by individual protective equipment and comprehensive measures, compared to the null scenario (Table 2). The QALYs of the comprehensive measures were higher than null, engineering controls, and individual protective equipment by 1.24, 0.1, and 0.75, respectively. Comprehensive measures and engineering controls are highly cost-effective compared with null. Individual protective equipment was cost-effective, with the ICER exceeding per capita GDP. However, taking engineering controls as the reference, comprehensive measures had an ICER of 454,691.90 RMB/QALY, which was over three times the per capita GDP. Individual protective equipment resulted in an additional cost of 7420.41 RMB, but QALYs decreased by 0.65 (ICER: -11,416.02 RMB/QALY), compared with engineering controls. The cost-effectiveness frontier is shown in Fig. 2. The results showed that engineering controls were the most cost-effective intervention, followed by comprehensive measures, and individual protective equipment dominated.

**Table 2:** Base case cost-effectiveness analysis

Interventions	Costs (RMB)	QALY	Incremental costs (RMB)	Incremental QALY	ICER (Costs/QALY)
Null	36,262.67	16.36	-	-	-
Engineering Controls	71,448.94	17.50	35,186.27	1.14	30,865.15
Individual Protective Equipment	78,869.35	16.85	42,606.68	0.49	86,952.41
Comprehensive Measures	116,918.13	17.60	80,655.46	1.24	65,044.73

### Sensitivity analyses

In one-way sensitivity analyses, changes in ICER when the costs and utilities were varied by 40% are shown in Table 3. Similar to the results of the base case analyses, the three interventions were cost-effective compared to the null scenario at the WTP threshold of three times the per capita GDP, except for individual protective equipment when the utility increased by 40%. To some extent, the results suggest the robustness of the base case analyses. Except when utility increased by 40%, the ICERs of engineering controls were less than the per capita GDP. In addition, when the cost increased or the cost of null decreased, the ICER of individual protective equipment exceeded the per capita GDP.

**Table 3:** One-way sensitivity analysis results

Variables	ICER(RMB/QALY)		
	**Engineering controls**	**Individual protective equipment**	**Comprehensive measures**
Utility			
-40%	18,716.10	53,258.35	39,344.13
+ 40%	90,221.21	236,703.78	192,036.81
Cost of null			
-40%	43,588.89	116,554.59	76,742.36
+ 40%	18,141.40	57,350.22	53,347.09
Cost of engineering controls			
-40%	5795.34	-	-
+ 40%	55,934.96	-	-
Cost of individual protective			
-40%	-	22,569.27	-
+ 40%	-	151,335.55	-
Cost of comprehensive measures			
-40%	-	-	27,329.20
+ 40%	-	-	102,760.26

**Fig. 3:**
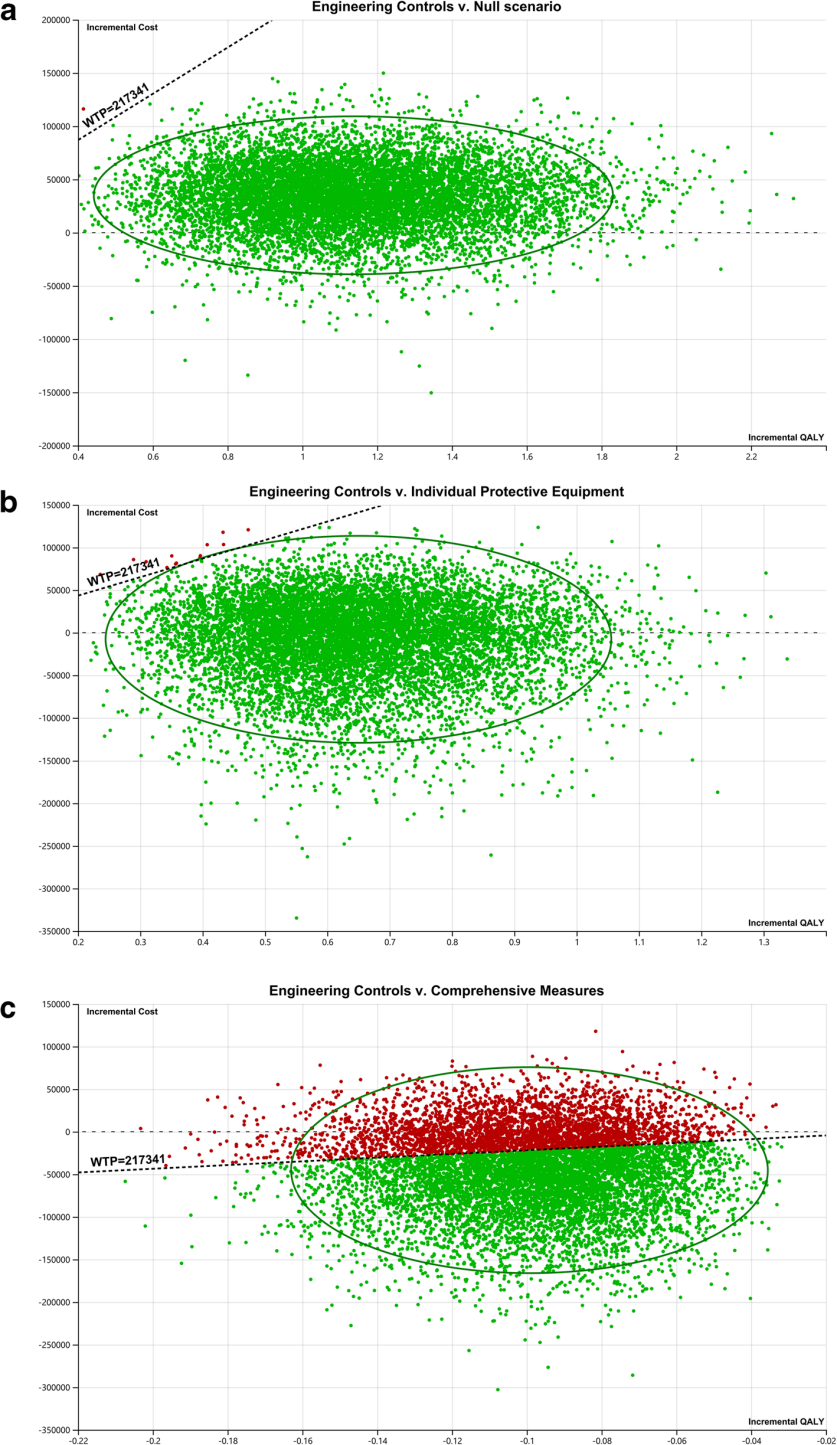
Incremental cost-effectiveness in 10,000 Monte Carlo simulations. **a** engineering controls *vs.* null scenario; **b** engineering controls *vs.* individual protective equipment; **c** engineering controls *vs.* comprehensive measures

**Fig. 4:**
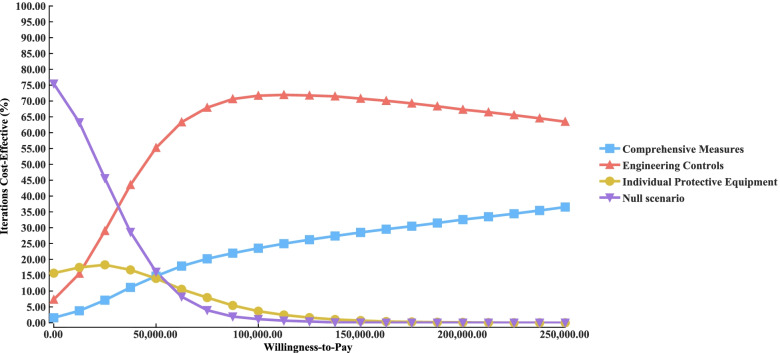
Acceptability curve for the cost-effective probabilities

Figure 3 shows the results of the probabilistic sensitivity analyses comparing engineering controls with other interventions. A cost-effectiveness probability of 99.99% for engineering controls at the WTP threshold of three times the per capita GDP compared to the null scenario was revealed (Fig. 3a). The probability that engineering controls were more cost-effective than individual protective equipment was 99.86% (Fig. 3b). The cost-effectiveness probability for engineering controls was 66.17% compared with the comprehensive measures (Fig. 3c). As shown in the acceptability curves, when the WTP threshold exceeded approximately 30,000 RMB, engineering controls were the most cost-effective intervention, with comprehensive measures ranking second (Fig. 4).

## Discussion

In this study, we assessed the cost-effectiveness of CWP preventive measures from the perspective of coal enterprises. Comprehensive measures that combine dustproof engineering technology and individual protection in China’s state-owned mines are cost-effective. Engineering controls are more cost-effective than individual protection controls. Sensitivity analysis revealed that the results were robust.

Due to the lack of studies on the cost of CWP preventive measures, we collected data from six state-owned coal mines. Generally, preventive measures vary from mine to mine, but the homogeneity of state-owned mines is better than that of private and township mines because of strict administration and supervision. Therefore, we conducted this study without considering private mines and township mines. Notably, the cost of individual protective equipment was higher than that of engineering prevention. This might be associated with the extensive usage and high replacement frequency of individual protective equipment during the mining process.

Multiple interrelated interventions play the role of a comprehensive integrated system in preventing CWP. For example, the health effect of wearing a dust mask is likely to depend on whether the dustproof equipment of spraying or ventilation is running. Optimising health resource allocation requires an understanding of the effectiveness of each intervention independently and in combination [19]. Therefore, we adopted the back-adjusting method to extract the hypothetical null scenario, engineering controls, and individual protective equipment. Occupational health administration and surveillance usually affect the development of CWP indirectly, unlike engineering measures or individual protective equipment that directly alter the extent of miners’ exposure to dust. It is difficult to evaluate the efficiency of these indirect interventions to prevent CWP; therefore, this was not considered in this study. Dustproof engineering and technology can effectively reduce the exposure of miners to dust in the workplace [28–31]. Since 2002, a series of laws and rules for dustproof device technical parameters and dust concentration limits in the workplace have been introduced in China. Therefore, we assumed a relatively high coverage and efficiency of 95% for the engineering controls. For individual protection, although the filter efficiency of dust masks for particulate matter is considered to be at least 90%, the actual efficiency is not ideal because of the influence on breathing, fitness on the face, comfort, leakage, and inadequate supervision [32–34]. A recent study showed that due to poor quality and inappropriate usage, the actual filtration efficiency and inward leakage of dust masks were 46.01% and 30.8%, respectively [35]. Therefore, a lower coverage and efficiency of 70% for individual protective equipment was assumed in the present study.

Our results showed that the QALYs of the comprehensive measures were the highest. However, the QALYs were only 0.1 more than the engineering controls with the cost greatly increased. Therefore, the engineering controls were more cost-effective than the comprehensive measures. The results also showed that the ICER of individual protective equipment exceeded the per capita GDP compared to the null. However, compared to engineering controls, individual protection was the dominated strategy. The results of the sensitivity analyses were consistent with the base case analyses, demonstrating the robustness of the results. As the model’s inputs varied, the ICERs of the comprehensive measures were less than three times the per capita GDP. Whether the inputs increased or decreased by 40%, the ICERs of engineering controls were less than the per capita GDP, except when utility increased to 0.93 (by 40%). To some extent, the results showed that engineering controls had absolute economic advantages. Individual protection equipment are not attractive compared to engineering measures. In our previous study, the opinions of 41 experts on occupational diseases were collected to create an index system for evaluating the quality of comprehensive measures against CWP [17]. The study identified that the weight of the dustproof engineering technology was twice that of individual protection, which was consistent with the present study to some extent. Our results suggest that coal enterprises should increase their investment in dustproof engineering and technology to improve the prevention of CWP. However, we do not deny the importance of individual protection, which is the last line of defence against CWP. We regarded the low cost-effectiveness of individual protection as a result of the low efficiency and coverage rate. Therefore, enhancing efficiency and coverage may be the core issue for improving individual protection. Therefore, it is necessary to develop dust masks with better fitness, comfortable capability, lower respiratory resistance, and leakage. In addition, training and education for coal miners and strict regular supervision are also needed.

This study had some limitations. First, because of the lack of large-scale cohort studies on CWP, we extracted the probabilities from different researches. Data from different sources may be subject to an overestimation or underestimation of cost-effectiveness. The US National Institute for Occupational Safety and Health administered the Coal Workers’ Health Surveillance Program. Free periodic chest radiographs were provided for coal miners working underground. However, participation is only in the 30%–40% range; hence, the true incidence and mortality of CWP are unclear [36, 37]. Thus, more large-scale cohort studies on coal miners are required. Second, the parameters used in the model were mainly Chinese state-owned enterprises. Therefore, the findings of this study can be extrapolated to other coal enterprises with care. In addition, due to the lack of studies on the utility score of Chinese coal workers after an extensive literature review, we extracted data from a Chinese study. The utility was similar to the results of a Mongolian survey of patients with occupational pneumoconiosis, in which it was 0.71 [38]. We performed one-way sensitivity analyses to explore the impact of the uncertain utility score on the ICER, and the results showed the robustness of the base case analyses.

## Conclusions

The comprehensive preventive measures for CWP that are currently implemented in Chinese state-owned coal mines are cost-effective. Furthermore, engineering measures are more cost-effective than individual protection measures. To improve the cost-effectiveness of preventing CWP, investment in engineering dustproof controls should be increased.

## Supplementary Information

**Additional file 1: Table S1.** Transition probabilities from health state to CWP for different interventions.

**Additional file 2: Table S2.** Transition probabilities in time-dependent Markov model.

## Data Availability

The datasets used and/or analysed during the current study available from the corresponding author on reasonable request.

## Abbreviations

CWP: Coal workers’ pneumoconiosis
RMB: Chinese Renminbi
GDP: Gross domestic product
WTP: Willingness-to-pay
QALYs: Quality-adjusted life years
ICERs: Incremental cost-effectiveness ratios
WHO: World Health Organization
GCEA: Generalised cost-effectiveness analysis
