# Supplementary material for: Cost-effectiveness of comprehensive preventive measures for coal workers’ pneumoconiosis in China
Source: BMC Health Serv Res. 2022 Feb 28;22:266. doi: 10.1186/s12913-022-07654-7 (PMC8883714; doi:10.1186/s12913-022-07654-7)
Supplement: Supplementary file 1 — Additional file 1: Table S1. Transition probabilities from health state to CWP for different interventions. [file 12913_2022_7654_MOESM1_ESM.docx]

**Additional file 1**

**Table S1: Transition probabilities from health state to CWP for different interventions**

| **Age** | **Interventions** | | | |
| --- | --- | --- | --- | --- |
|  | **Comprehensive measures** | **Engineering controls** | **Individual protective equipment** | **Null** |
| 20 | 0.00000 | 0.00000 | 0.00000 | 0.00000 |
| 21 | 0.00000 | 0.00000 | 0.00000 | 0.00000 |
| 22 | 0.00001 | 0.00002 | 0.00012 | 0.00023 |
| 23 | 0.00000 | 0.00000 | 0.00000 | 0.00000 |
| 24 | 0.00001 | 0.00002 | 0.00012 | 0.00024 |
| 25 | 0.00002 | 0.00005 | 0.00025 | 0.00049 |
| 26 | 0.00002 | 0.00005 | 0.00026 | 0.00050 |
| 27 | 0.00027 | 0.00052 | 0.00273 | 0.00536 |
| 28 | 0.00009 | 0.00018 | 0.00093 | 0.00182 |
| 29 | 0.00032 | 0.00062 | 0.00323 | 0.00634 |
| 30 | 0.00045 | 0.00089 | 0.00464 | 0.00910 |
| 31 | 0.00106 | 0.00207 | 0.01085 | 0.02128 |
| 32 | 0.00090 | 0.00177 | 0.00924 | 0.01812 |
| 33 | 0.00127 | 0.00249 | 0.01300 | 0.02550 |
| 34 | 0.00149 | 0.00292 | 0.01530 | 0.03000 |
| 35 | 0.00245 | 0.00481 | 0.02518 | 0.04937 |
| 36 | 0.00177 | 0.00348 | 0.01820 | 0.03568 |
| 37 | 0.00243 | 0.00476 | 0.02488 | 0.04879 |
| 38 | 0.00139 | 0.00273 | 0.01426 | 0.02797 |
| 39 | 0.00235 | 0.00460 | 0.02406 | 0.04718 |
| 40 | 0.00232 | 0.00456 | 0.02383 | 0.04673 |
| 41 | 0.00155 | 0.00304 | 0.01588 | 0.03114 |
| 42 | 0.00196 | 0.00384 | 0.02009 | 0.03939 |
| 43 | 0.00264 | 0.00517 | 0.02706 | 0.05305 |
| 44 | 0.00334 | 0.00654 | 0.03421 | 0.06708 |
| 45 | 0.00282 | 0.00554 | 0.02896 | 0.05679 |
| 46 | 0.00418 | 0.00820 | 0.04291 | 0.08414 |
| 47 | 0.00402 | 0.00788 | 0.04122 | 0.08082 |
| 48 | 0.00503 | 0.00987 | 0.05164 | 0.10125 |
| 49 | 0.00484 | 0.00948 | 0.04961 | 0.09727 |
| 50 | 0.00544 | 0.01066 | 0.05577 | 0.10935 |
| 51 | 0.00559 | 0.01096 | 0.05732 | 0.11239 |
| 52 | 0.00694 | 0.01361 | 0.07121 | 0.13963 |
| 53 | 0.00751 | 0.01472 | 0.07698 | 0.15094 |
| 54 | 0.00843 | 0.01652 | 0.08641 | 0.16943 |
| 55 | 0.01134 | 0.02223 | 0.11631 | 0.22805 |
| 56 | 0.00933 | 0.01829 | 0.09569 | 0.18763 |
| 57 | 0.00779 | 0.01527 | 0.07988 | 0.15663 |
| 58 | 0.00757 | 0.01483 | 0.07759 | 0.15214 |
| 59 | 0.00933 | 0.01829 | 0.09569 | 0.18763 |
| 60 | 0.02545 | 0.04990 | 0.26103 | 0.51183 |
| 61 | 0.02418 | 0.04740 | 0.24796 | 0.48619 |
| 62 | 0.00000 | 0.00000 | 0.00000 | 0.00000 |
| 63 | 0.03390 | 0.06647 | 0.34767 | 0.68172 |
