# Supplementary material for: Cost-effectiveness of comprehensive preventive measures for coal workers’ pneumoconiosis in China
Source: BMC Health Serv Res. 2022 Feb 28;22:266. doi: 10.1186/s12913-022-07654-7 (PMC8883714; doi:10.1186/s12913-022-07654-7)
Supplement: Supplementary file 2 — Additional file 2: Table S2. Transition probabilities in time-dependent Markov model. [file 12913_2022_7654_MOESM2_ESM.docx]

**Additional file 2**

**Table S2: Transition probabilities in time-dependent Markov model**

| **Age** | **Transition probabilities** | | | |
| --- | --- | --- | --- | --- |
|  | **Health to death** | **Health to health** | **CWP to death** | **CWP to CWP** |
| 20 | 0.00047 | 0.99953 | 0.00082 | 0.99918 |
| 21 | 0.00047 | 0.99953 | 0.00082 | 0.99918 |
| 22 | 0.00050 | 0.99949 | 0.00082 | 0.99918 |
| 23 | 0.00054 | 0.99946 | 0.00082 | 0.99918 |
| 24 | 0.00056 | 0.99943 | 0.00082 | 0.99918 |
| 25 | 0.00058 | 0.99939 | 0.00082 | 0.99918 |
| 26 | 0.00057 | 0.99940 | 0.00082 | 0.99918 |
| 27 | 0.00059 | 0.99914 | 0.00082 | 0.99918 |
| 28 | 0.00061 | 0.99930 | 0.00082 | 0.99918 |
| 29 | 0.00068 | 0.99900 | 0.00082 | 0.99918 |
| 30 | 0.00070 | 0.99885 | 0.00167 | 0.99833 |
| 31 | 0.00077 | 0.99817 | 0.00167 | 0.99833 |
| 32 | 0.00081 | 0.99829 | 0.00167 | 0.99833 |
| 33 | 0.00083 | 0.99790 | 0.00167 | 0.99833 |
| 34 | 0.00094 | 0.99756 | 0.00167 | 0.99833 |
| 35 | 0.00103 | 0.99652 | 0.01006 | 0.98994 |
| 36 | 0.00106 | 0.99716 | 0.01006 | 0.98994 |
| 37 | 0.00114 | 0.99644 | 0.01006 | 0.98994 |
| 38 | 0.00121 | 0.99740 | 0.01006 | 0.98994 |
| 39 | 0.00134 | 0.99631 | 0.01006 | 0.98994 |
| 40 | 0.00151 | 0.99617 | 0.01757 | 0.98243 |
| 41 | 0.00155 | 0.99690 | 0.01757 | 0.98243 |
| 42 | 0.00182 | 0.99622 | 0.01757 | 0.98243 |
| 43 | 0.00189 | 0.99547 | 0.01757 | 0.98243 |
| 44 | 0.00207 | 0.99460 | 0.01757 | 0.98243 |
| 45 | 0.00231 | 0.99486 | 0.00700 | 0.99300 |
| 46 | 0.00236 | 0.99346 | 0.00700 | 0.99300 |
| 47 | 0.00254 | 0.99344 | 0.00700 | 0.99300 |
| 48 | 0.00311 | 0.99185 | 0.00700 | 0.99300 |
| 49 | 0.00328 | 0.99188 | 0.00700 | 0.99300 |
| 50 | 0.00364 | 0.99092 | 0.01513 | 0.98487 |
| 51 | 0.00375 | 0.99066 | 0.01513 | 0.98487 |
| 52 | 0.00398 | 0.98908 | 0.01513 | 0.98487 |
| 53 | 0.00441 | 0.98808 | 0.01513 | 0.98487 |
| 54 | 0.00498 | 0.98659 | 0.01513 | 0.98487 |
| 55 | 0.00518 | 0.98348 | 0.00296 | 0.99704 |
| 56 | 0.00564 | 0.98503 | 0.00296 | 0.99704 |
| 57 | 0.00609 | 0.98612 | 0.00296 | 0.99704 |
| 58 | 0.00681 | 0.98563 | 0.00296 | 0.99704 |
| 59 | 0.00767 | 0.98300 | 0.00296 | 0.99704 |
| 60 | 0.00854 | 0.96601 | 0.00489 | 0.99511 |
| 61 | 0.00938 | 0.96645 | 0.00489 | 0.99511 |
| 62 | 0.01038 | 0.98962 | 0.00489 | 0.99511 |
| 63 | 0.01112 | 0.95498 | 0.00489 | 0.99511 |
